# Modulation of the Intratumoral Immune Landscape by Oncolytic Herpes Simplex Virus Virotherapy

**DOI:** 10.3389/fonc.2017.00136

**Published:** 2017-06-26

**Authors:** Jie Yin, James M. Markert, Jianmei W. Leavenworth

**Affiliations:** ^1^Department of Neurosurgery, University of Alabama at Birmingham, Birmingham, AL, United States; ^2^Department of Microbiology, University of Alabama at Birmingham, Birmingham, AL, United States; ^3^Department of Pediatrics, University of Alabama at Birmingham, Birmingham, AL, United States; ^4^Department of Cell, Developmental and Integrative Biology, University of Alabama at Birmingham, Birmingham, AL, United States

**Keywords:** oncolytic virotherapy, herpes simplex virus, tumor microenvironment, immune crosstalk, innate immunity, adaptive immunity, metabolic programming, immunogenic cell death

## Abstract

Vaccines and immunotherapeutic approaches to cancers with the advent of immune checkpoint inhibitors and chimeric antigen receptor-modified T cells have recently demonstrated preclinical success and entered clinical trials. Despite advances in these approaches and combinatorial therapeutic regimens, depending on the nature of the cancer and the immune and metabolic landscape within the tumor microenvironment, current immunotherapeutic modalities remain inadequate. Recent clinical trials have demonstrated clear evidence of significant, and sometimes dramatic, antitumor activity, and long-term survival effects of a variety of oncolytic viruses (OVs), particularly oncolytic herpes simplex virus (oHSV). Acting as a multifaceted gene therapy vector and potential adjuvant-like regimens, oHSV can carry genes encoding immunostimulatory molecules in its genome. The oncolytic effect of oHSV and the inflammatory response that the virus stimulates provide a one-two punch at attacking tumors. However, mechanisms underlying oHSV-induced restoration of intratumoral immunosuppression demand extensive research in order to further improve its therapeutic efficacy. In this review, we discuss the current OV-based therapy, with a focus on the unique aspects of oHSV-initiated antiviral and antitumor immune responses, arising from virus-mediated immunological cell death to intratumoral innate and adaptive immunity.

## Introduction

**Figure 1:**
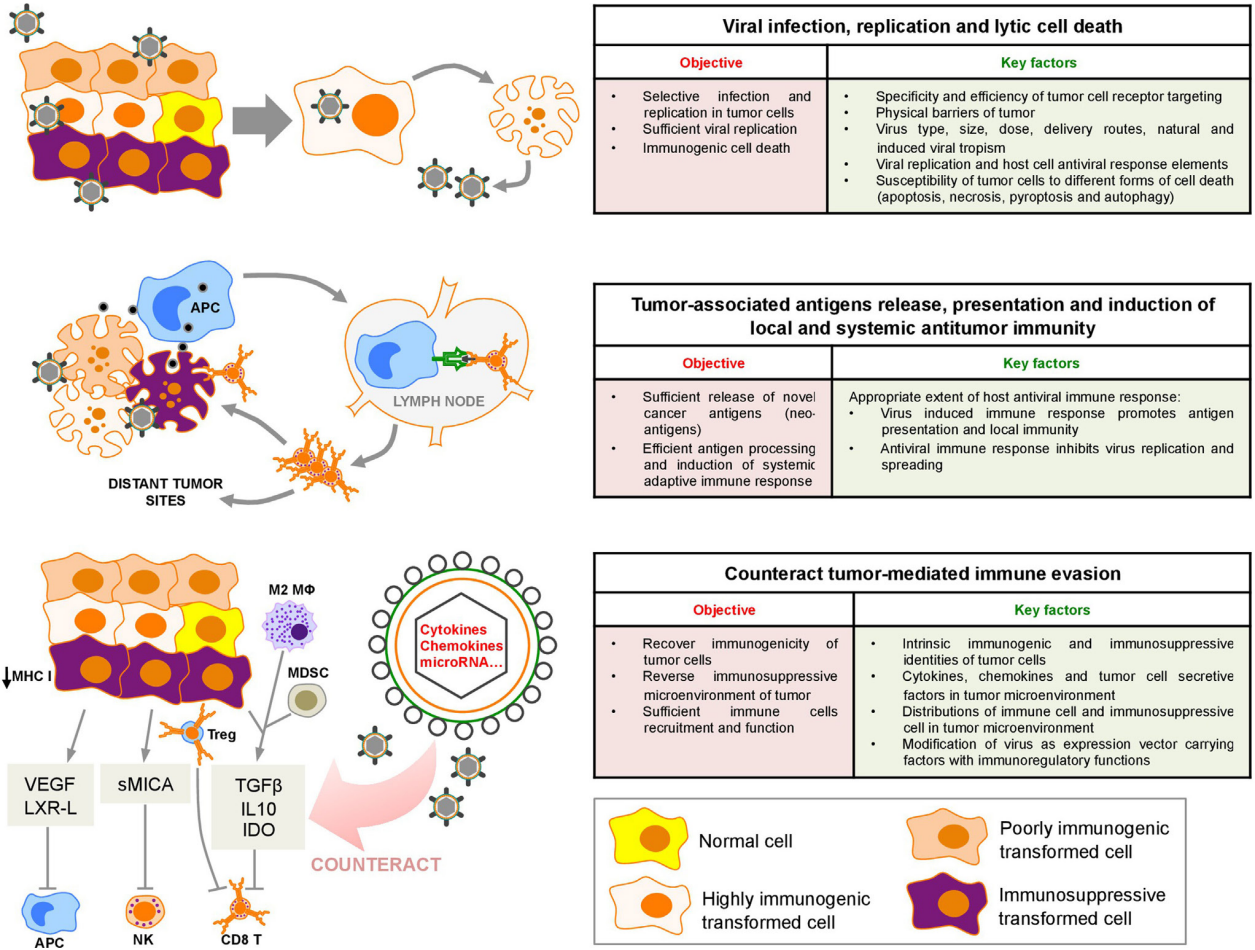
Sequential oncolytic herpes simplex virus-induced events: virus infection, cell death, and innate and adaptive immune responses within the tumor microenvironment. VEGF, vascular endothelial growth factor; LXR-L, liver X receptor ligand; sMICA, soluble MHC class I polypeptide-related sequence A; TGF-β, transforming growth factor-β; IL10, interleukin 10; IDO, indoleamine 2,3-deoxygenase; APC, antigen-presenting cell; M2 MΦ, M2 macrophages; MDSC, myeloid-derived suppressor cells; Treg, regulatory T cell; NK, natural killer.

The various cellular subsets within the tumor, including cancer cells, stromal cells, and infiltrating immune cells, interplay and contribute to a highly immunosuppressive microenvironment. Cancer cells undergoing stochastic genetic and epigenetic changes generate the critical modifications necessary to circumvent both innate and adaptive immunological defenses. Tumors evade immunity by downregulating antigen presentation, upregulating inhibitors of apoptosis, or expressing inhibitory surface molecules (e.g., programmed death-ligand 1) (1). In addition, tumor cells secrete factors [e.g., transforming growth factor beta (TGF-β), indoleamine 2,3-dioxygenase (IDO)] that directly inhibit effector immune cell functions or recruit regulatory cells, tumor-associated macrophages, and myeloid-derived suppressor cells (MDSC) to intensify an immunosuppressive microenvironment (Figure 1) (1). The specific intratumoral immune landscape within a certain type of cancer further contributes to tumor progression by selecting more aggressive tumor variants. In light of the importance of immune regulation in tumor growth, cancer immunotherapeutic approaches, aimed to interfere with tumor immunosuppressive microenvironment and boost antitumor immune responses, have emerged as promising strategies. Among these approaches, checkpoint inhibitors [PD-1 and cytotoxic T-lymphocyte-associated protein 4 (CTLA-4) antibodies] have been successfully used to treat several types of cancers (2, 3). However, only limited numbers of cancer patients show remission after treatment (2), indicating a pivotal effect of heterogeneous immune background on the outcome of immunotherapy, and suggesting that alternative or combined immunotherapeutic strategies should be considered.

Since the discovery of the oncolytic effect of virus infection a century ago, oncolytic virotherapy with a variety of viruses, including wild-type viruses, attenuated viruses and transgenic viruses, has emerged as a potential therapeutic approach to treat cancer (4). To date, OVs based on 11 DNA and RNA virus platforms are actively tested in clinical trials (5). The most successful one is the talimogene laherparepvec (T-VEC) derived from the herpes simplex virus (HSV), which has finished the Phase III clinical trial and been approved for the treatment of advanced metastatic melanoma in 2015 by US Food and Drug Administration (6).

Herpes simplex virus-1 is a double-stranded DNA virus possessing a large and well-characterized genome (152 kb), and about 30 kb is dispensable for viral infection. This unique feature makes HSV-1 suitable for genetic manipulation. In addition, although HSV-1 replicates in the nucleus, it does not cause insertional mutagenesis and is sensitive to aciclovir and ganciclovir (7). These safety features make HSV-1 an attractive candidate for oncolytic virotherapy. Besides T-VEC, we and others have developed several other oncolytic herpes simplex viruses (oHSVs) that have proceeded into clinical trials, for example, G207, an HSV-1 mutant with deletions of both copies of γ_1_34.5 gene encoding the infected-cell protein 34.5 (ICP34.5) and a lacZ insertion into the UL39 neurovirulence gene (8); HSV1716, a γ_1_34.5 null mutant with an intact UL39 gene that replicates selectively in actively dividing cells; and G47Δ, which is built from G207 by the deletion of the α47 gene (9). These oHSVs have been evaluated in multiple tumor types in murine models and patients (10–12). In particular, the neurotropic feature of oHSV makes it an attractive option for brain cancer therapy (7).

oHSVs mediate antitumor activity through direct lysis of tumor cells and the subsequent induction of systemic antitumor immunity. The induction of antitumor immune reaction is pivotal for the effect of oHSV therapy (13). We have recently reviewed the oHSV-based therapy for malignant glioma (7). Here, we focus on the sequences of immune responses to such therapy (Figure 1) and provide insight into how we can utilize these information to improve this therapy and/or combine with other approaches to increase the oHSV antitumor efficacy.

## Intratumoral Immune Landscape

The immune system is capable of recognizing tumors and eliminates early malignant cells. Nonetheless, cancer progression ultimately escapes immune-mediated destruction. Based on biopsies and gene profiling analysis of various types of tumor samples from individual patients, accumulating evidence shows that there are two distinct subsets of patients (14). One subset of patients shows evidence of spontaneous T-cell priming and immune infiltration into tumors. This phenotype has been characterized as the T cell-inflamed tumor microenvironment with the expression of various T-cell transcripts and chemokines that likely mediate T-cell recruitment, antigen-presenting cell (APC) activation, and a type I interferon (IFN) signature (14). Immunohistochemical analysis has confirmed the presence of CD8^+^ T-cells, macrophages, some B-cells, and plasma cells in these tumors (15). In contrast to this spontaneous immune activation, the non-T cell-inflamed tumors lack all of these parameters and are devoid of T-cells (15). The characteristics of these two distinct phenotypes have suggested two broad categories of tumor evasion of host immunity. In T cell-inflamed tumors, immune failure appears to occur at the effector phase, and some patients with this type of tumor show good clinical responses to cancer vaccines, high-dose interleukin (IL)-2, anti-CTLA-4, and anti-PD-1 antibodies (16–18). Non-T cell-inflamed tumors suggest immune exclusion (15), and the current wave of immunotherapies being explored clinically seems unlikely to be successful in these cases. Further characterization of the immune contexture of individual tumors based on the tumor genomic landscape, extent of DNA damage, mutational load, and neoantigen presentation may direct more efficient approaches and better prediction of therapeutic responses (19).

All cells, including cancer and immune cells, need to produce ATP through oxidative metabolism and synthesize macromolecules through glycolysis and/or glutaminolysis to maintain their basic cellular functions (20, 21). Tumor cell proliferation and growth depend on glycolysis and glutaminolysis, a hallmark of cancer metabolism (20, 22). Metabolites secreted from tumors alter the microenvironment, enable tumors to adapt to hypoxia, and also regulate intratumoral immune cells. Metabolic pathways of oxidative metabolism, glycolysis, and glutaminolysis preferentially fuel the cell fate decisions and effector functions of all immune cells (21). Immune cells can rapidly shift between glycolysis and oxidative phosphorylation in response to external signals, which is important for their development, activation, and normal function (21, 23). Although the metabolic regulation of immune cells is not the focus of this review and has been extensively reviewed by others (21), it should be noted that complex metabolic interactions between stromal cells, cancer cells, and immune cells in the microenvironment can promote tumor growth and suppress immune reactions. Tumor cells with high metabolic demand may compromise the function of some immune cells by competing glucose and other nutrients, leading to T-cell dysfunction such as anergy and exhaustion and may also support the function of immunosuppressive cells by forming a metabolic symbiosis. Future immunotherapeutic approaches to reprogramming the metabolic pathways of immune cells and normalizing the intratumoral immune landscape should be considered.

## Intratumoral oHSV Replication and Induction of Immunogenic Cell Death (ICD)

OVs preferentially accumulate and replicate in tumor cells with aberrant apoptosis, proliferation, and antiviral signaling pathways. In normal healthy cells, double-stranded viral RNA and other viral elements can be recognized by protein kinase R (PKR), which is a component of intracellular antiviral machinery (24). Activated PKR phosphorylates eukaryotic initiation factor (eIF2α), leading to cell protein synthesis termination and rapid cell death. Wild-type HSV escapes antiviral response due to expression of the ICP34.5 protein which activates a phosphatase that then dephosphorylates eIF2α, restoring protein synthesis in the infected cell (25). Another important antiviral mechanism is mediated by intracellular toll-like receptors (TLRs) that recognize virus-related pathogen-associated molecular patterns (PAMPs) and subsequently induce local IFN release (26, 27). In cancer cells, abnormal IFN pathway and PKR activity promote tumor-specific replication of oHSV. Attenuated oHSVs, including G207 and HSV1716, are depleted of ICP34.5, which render oHSV unable to block PKR phosphorylation, resulting in preferential lysis of tumor cells compared to normal cells (7). oHSVs can also mediate targeted lysis of cancer stem cells (CSCs) (28). These cells are rare populations of tumor-initiating cells that are capable of self-renewal and have pluripotent capacity (29). CSCs are particularly resistant to chemotherapies and radiation therapies, making them the primary source of drug resistance, metastasis, and tumor recurrence. The efficacy and potential of oHSV in targeting CSCs have been extensively discussed previously (30). We have recently found that xenografts of pediatric medulloblastoma CSCs are highly sensitive to killing by oHSVs G207 or M002, a neuroattenuated oHSV expressing murine IL-12 (31).

Replication of OVs in tumor cells can induce different types of cell death including necrosis, apoptosis, pyroptosis, and autophagic cell death. Depending on the initiating stimulus, cancer cell death can be immunogenic or non-immunogenic (32). ICD involves changes in the composition of the cell surface as well as the release of soluble mediators, which operate on a series of receptors expressed by dendritic cells (DC) to stimulate T-cells (33). Cancer cells undergoing ICD expose calreticulin (CRT) on the outer leaflet of their plasma membrane followed by a sequential secretion of ATP and high mobility group box 1 (HMGB1) (33). ATP, CRT, and HMGB1 bind to their respective receptors on immature DCs to facilitate the recruitment of DCs into the tumor bed, the engulfment of tumor antigens by DCs, and optimal antigen presentation to T-cells (32). ICD constitutes a prominent pathway for the activation of antitumor immunity, which involves release of danger-associated molecular patterns (DAMPs) and tumor-associated antigens (TAAs). By inducing ICD of tumor cells, OVs facilitate TAAs cross-presentation to DCs and finally induce antitumor immune responses. A recent study conducted with squamous cell carcinoma cells shows efficient ICD after oHSV infection (34). ICD is the mainstay of long-term success for anticancer therapies, and it may also hold promise for developing oHSVs as potential cancer vaccines or adjuvants for these vaccines.

## Innate Immunity in oHSV Therapy

The generation of a robust adaptive immune response against cancer must, in principle, rely on upstream innate immune activation that leads to productive T-cell priming. In a non-T cell-inflamed tumor, restoring dysfunctional innate immunity is the key point of new therapeutic interventions. Here, we focus on the innate immune responses mediated by NK cells and DCs.

OV-induced cancer cell death releases PAMPs or DAMPs that are recognized by pattern recognition receptors, such as TLRs, located in the cytoplasm or on the cell surface. Their engagement induces expression of inflammatory cytokines (e.g., IFNs, tumor necrosis factor-α, IL-6, and IL-12), which bind to receptors on other cells, resulting in recruitment and activation of innate immune cells, such as NK, NKT, and γδ T-cells (5, 7). NK cells have been recognized as a relevant first-line defense against viruses. NK cells can sense infected cells either through direct interaction with PAMPs *via* TLRs or through recognition of viral and/or virus-induced ligands *via* activating NK cell receptors (35). Upon activation NK cells directly kill infected cells through cytotoxicity or boost immune responses *via* cytokine secretion. NK cells may exert either positive or negative effects on oHSV therapy, depending on several factors such as virus type, dose, and replication rate (36, 37). An optimal balance of NK activating and inhibiting signals may be particularly relevant for oHSV-based therapies. Alvarez-Breckenridge et al. have elegantly demonstrated that HSV-induced upregulation of the ligands for natural cytotoxic receptors triggers NK cells to mediate premature clearance of oHSV in a mouse glioblastoma model, suggesting a potential limitation in glioblastoma virotherapy (38). In contrast, studies using UV-inactivated HSV suggest that the surface components of UV-HSV directly activate NK cells and enhance NK-cell killing of leukemia cells (39).

One of the important immune cells that bridge innate and adaptive immune responses is the DC. DCs are classically divided into two major categories: plasmacytoid DCs (pDCs) and conventional DCs (cDCs) (40). pDCs are specialized in the secretion of high levels of type I IFNs upon stimulation *via* TLRs. Within the cDC compartment, the CD8α^+^ DC subtype is most efficient at phagocytosing dead cells and in cross-presenting antigens to CD8^+^ T-cells (40). Sufficient production of type I IFNs by APC, including DCs, in the tumor microenvironment is critical for induction of adaptive antitumor T-cell responses. Tumors absent of type I IFN signature usually respond poorly to conventional immunotherapies (41). The stimulator of interferon genes (STING) is a key cytosolic DNA sensor for the detection of intracellular pathogens, notably DNA viruses like HSV (42, 43). DNA released from dying tumor cells can be sensed by the cytosolic enzyme cyclic GMP-AMP synthase (cGAS). Cyclic dinucleotides generated by cGAS bind to STING and induce type I IFN production through phosphorylation of interferon regulatory factor 3 (40). Xia et al. have provided evidence that STING is frequently functionally suppressed in human cancers. Loss of STING prevents DNA damage-mediated type I IFN production, which renders tumor cells highly susceptible to OV infection (44), suggesting that STING activity might be a crucial indicator to stratify cancer patients for OV-based therapies.

## Adaptive Immunity in oHSV Therapy

Sufficient innate immune responses lead to APC maturation and antigen presentation to naïve T-lymphocytes, which activates antigen-specific CD4^+^ helper T (T_H_)-cells and CD8^+^ effector T-cells. Once activated, these T-cells expand and traffic to tumor sites, where they mediate antitumor immunity. Although priming adaptive immunity plays a critical role in OV-mediated antitumor activity, the natural ability of viruses to induce host antiviral immune responses may result in clearance of the virus through neutralizing antiviral antibodies and/or cytotoxic T-cell-mediated immune responses (5). The extent to which viral neutralization influences the induction of antitumor immunity is complex and can be influenced by many variables, most notably the characteristics of the virus and the tumor microenvironment. For example, HSV-1 evades CD8^+^ T-cells by producing ICP47, which limits immune recognition of infected cells by inhibiting the transporter associated with antigen processing (TAP) (45). An engineered oHSV carrying a bovine herpesvirus homologous gene of ICP47 shows superior efficacy in treating bladder and breast cancer in murine models, which is dependent upon CD8^+^ T-cells (46), suggesting that arming oHSVs with TAP inhibitor may enhance local and systemic antitumor responses.

Unlike innate immunity, the adaptive immune response generates immune memory, implying that any subsequent exposure to the same antigen that immune cells encounter previously will induce a stronger response. When using OV therapy, the antiviral memory response must be taken into consideration because it prevents retreatment, which is an essential component of OV-based therapy (4, 47). Humans are naturally (or artificially through vaccination) exposed to HSV and may therefore have preexisting neutralizing antibodies or cellular immunity against HSV. Strategies to limit virus neutralization include utilizing alternative virus serotypes or developing wild-type, non-human viruses. However, OV-induced immune memory to tumor antigens due to epitope spreading is an integral immune component of OV therapy (5). This is exemplified by the finding that immunocompetent mice treated with a parvovirus OV do not develop glioma and long-term survivors fail to develop tumors when rechallenged with uninfected tumor cells (48). Antitumor memory response is also essential for the development of tumor vaccines. Therefore, understanding mechanisms for the generation of antitumor memory responses is required for designing strategies to enhance OV and oHSV therapies.

## Improving Therapy: Modified oHSV and Combinatorial Therapy

OVs revive the suppressive microenvironment through a variety of mechanisms that alter the cytokine milieu and the type of immune cells within the tumor (5). Clinical efficacy can be increased by modifying the viral backbone or by developing OVs with multimodal activity. An extensive panel of transgenes, including inflammatory cytokines, antiangiogenic and antivascular proteins, monoclonal antibodies, proapoptotic genes, and enzymes that degrade extracellular matrix, have been used to modify the oHSV backbone to enhance their therapeutic efficacy in preclinical and clinical studies. The oHSV T-VEC is armed with human granulocyte-macrophage colony-stimulating factor, an inflammatory cytokine that bolsters antitumor immune responses by recruiting NK cells and inducing TAA-specific cytotoxic T-cells (49). oHSVs armed with other cytokines (e.g., IL-2, IL-12, IL-15, IL-18, and IFN-α/β), chemokines (e.g., CCL5), or costimulatory molecules (e.g., B7.1 and CD40L) can also induce antitumor immunity (50). For instance, an oHSV armed with IL-12, a potent antitumor cytokine with antiangiogenic activities, reduces neovasculature and Tregs, and induces T_H_1-mediated immunity in an immunocompetent CSC model (51). We have developed a neuroattenuated oHSV expressing human IL-12, termed M032, which is currently in Phase I clinical trial on patients with recurrent gliomas.

Combinatorial therapy using drugs or distinct immunomodulatory methods with oHSV to activate the immune response and/or block the immunosuppressive tumor microenvironment also has great potential to improve the overall clinical efficacy. Combinatorial therapy regimens that circumvent intracellular and microenvironmental antiviral responses are good options. Depending on the cancer type, tumor immunogenicity, and tumor microenvironment, OVs can be combined with approved immunoregulatory approaches, including epigenetic modifiers (e.g., histone deacetylase inhibitors, DNA methylation inhibitors, and histone methyltransferase inhibitors) (52–54), adoptive T-cell transfer therapy (e.g., chimeric antigen receptor T-cell therapy) (55), immune checkpoint inhibitors (antibodies targeting CTLA-4, PD-1, lymphocyte-activation gene 3, or T-cell immunoglobulin and mucin-domain containing-3) (56–59), activation of stimulatory pathways (antibodies targeting CD137, OX-40, and inducible T-cell costimulator) (60, 61), targeting suppressive mechanisms in the microenvironment (IDO and TGF-β inhibitors) (62–64), novel multifunctional immunoregulatory targets (e.g., osteopontin) (65, 66), and chemotherapeutic drugs (e.g., gemcitabine, 5-fluorouracil, and retinoic acid) that delete immunosuppressive cells (Tregs, MDSCs, and M2 macrophages) (67–69). Promising results have been obtained when OVs are combined with an antibody that blocks T-cell checkpoint inhibitory receptors, such as CTLA-4 or PD-1 (56, 57). However, successful combinatorial therapy is context dependent, and additional studies are needed to define the optimal therapeutic conditions.

## Conclusion

By virtue of its safety and suitability for genetic manipulation as a multifaceted gene therapy vector, oHSV-based therapy has emerged as a promising cancer immunotherapeutic approach. It may be particularly desirable for those non-T cell-inflamed tumors that are refractory to other immunotherapies. oHSV infection not only lyses the tumor but also induces cytokine production and immune cell recruitment into tumors, which reinvigorate the immunosuppressive environment and may restore the metabolic landscape within the tumors. Although promising results have been obtained using oHSV alone or combined with other approaches on several types of cancers, challenges remain regarding how to improve the therapeutic outcomes by simultaneously maximizing both oHSV replication and antitumor immune responses. Additional studies are also needed to determine if oHSV can be combined with metabolic interventions to adjust the metabolic interplay within the tumor, how to sustain the oHSV-induced responses, particularly memory responses, and how to develop it as a cancer vaccine or adjuvant for current tumor-targeted DC vaccines. A more complete understanding of the crosstalk between tumor and immune system will guide the development of optimal interventions on cancer without compromising antitumor immunity.

## Author Contributions

JY, JM, and JL drafted the manuscript, revised it critically, and approved this final version for publication.

## Conflict of Interest Statement

The authors declare that research was conducted in the absence of any commercial or financial relationships that could be construed as a potential conflict of interest. JM was a co-founder of and owned stock and stock options (<8%) in Catherex Inc., a biotechnology company that had licensed additional intellectual property related to oHSV. Catherex Inc. was sold to Amgen Inc. in a structured buyout on December 18, 2015, and he no longer participates in any decision making or has any control of any aspect of Catherex or Amgen, although he did receive proceeds from the sale of the company. He is also a co-founder of and holds stock in another OV-related company, Aettis Inc.
